# The Cure of Consumption

**Published:** 1899-05-27

**Authors:** 


### The Cure of Consumption.

It has often been said that the difficulty of curing any malady may be estimated by the number of infallible remedies for it which from time to time have been brought forward, extolled, tested, abandoned, and forgotten. It would be impossible to find better illustrations of the truth of this statement than those which have been furnished by cholera among acute, and by pulmonary consumption among chronic diseases; and the latter now appears only too likely to be largely exploited by practitioners who are more eager to announce discoveries than to submit them to the test of careful and scientific observation. The identification of a bacillus as the immediate cause of tubercle has at once afforded a means of establishing the diagnosis of consumption with certainty, and of showing in what way and under what conditions the cause may be destroyed or rendered inoperative, so as to justify the assertion that a "cure" has been effected, and to place it beyond doubt that the traditional character of hopelessness need no longer be attached, at least to the early stages of the disease. But, as Robert Macaire says, "*Le jour va passer, mais les badauds ne passeront pas*," and the class of people who, a century ago, were ready to proclaim that consumption could be cured by living for six months in a cow byre, or who, fifty years ago, were eager to send their friends to die at Madeira, are now proclaiming that the only thing necessary is residence in a "sanatorium," and submission to the "open-air treatment." Others of similar mental character are proclaiming with equal ardour that Dr. This or Dr. That (usually a foreigner) has at last discovered the precise mixture of some volatile bactericide with atmospheric air which is required in order to kill the bacilli without interfering with the structure or the functions of the lungs; and that the veritable Bethesda is only to be found in the establishments in which the mixture in question is duly supplied to the inhabitants. From such announcements cruel disappointments arise, and there can be no question that valuable lives are sacrificed which, by greater discretion, might have been at least prolonged, and possibly preserved. A famous English surgeon of the sixteenth century, William Clowes, in his "Profitable and Necessary Book of Observations," tells his readers that four things are necessary to a cure—the grace of God, the obedience of the patient, the purity of the remedies, and the skill of the surgeon; and his wise counsel might at least serve to remind the credulous that the problems presented by departures from health are always complicated, and that the conditions of recovery must, therefore, be far less obvious and simple than is frequently believed. Any statement which is sufficiently opposed to common experience to be "startling" to the ignorant, and which admits of a few tawdry pictures of a house and its inmates and surroundings, is thought by some magazine editors to be "good copy"; and hence what is described as information about this or that "health resort" becomes freely distributed among people not unlikely to be influenced by it, and utterly incapable of estimating its actual value. Sometimes we find a writer who has passed a week in or near some "sanatorium," and who does not even know how to spell the technicalities which he has heard and tries to use, gravely describing to his readers the errors and shortcomings of "doctors" with regard to some form of illness which is supposed to be specially amenable to the particular baths, or inhalations, or diet, or waters, or modes of exercise, for which the sanatorium in question is famous, or which constitute the groundwork of its difference from competing establishments. Among all this chaotic chatter and babble, much of it proceeding from people whose business it is to subordinate truth to effect, the actual or supposed victim of consumption is liable to be sorely perplexed, and to be led into the oftentimes fatal mistake of thinking that he can decide for himself among the conflicting claims of the remedies and the localities which are pressed upon his attention. There is just one consideration, in relation to such conflicting claims, which the frequently overrated powers of "common sense" ought to be able to take into account. It is that all genuine medical knowledge, and all sound medical discovery speedily become the common property of all the doctors in the civilised world. Dr. A., in the next street, knows as much about the tubercle bacillus and the methods of destroying it as Dr. B. in Paris, or Dr. C. in the Black Forest, or Dr. D. in the Engadine, or Dr. E. on the Mediterranean; while about the patient, his actual condition, the prospects of recovery under any treatment, and his fitness to undergo this method or that, Dr. A. has from the beginning knowledge which the other learned letters of the alphabet, in the case of the particular person, can only acquire by the aid of opportunities for the observation of symptoms such as Dr. A. has already possessed, and of which he has already availed himself. The main advantage of a "sanatorium" which cannot be obtained elsewhere depends upon the greater willingness of the sick to submit to discipline, and to do what they are told, when they are removed from the influences, and, it may be, from the over indulgence, of friends at home. As far as this advantage is concerned it might surely be regarded as the province of "common sense" to secure it everywhere. Dr. A. has only to be sufficiently decided; and the patient and his family have only to remember that "the chamber of the sick man is the kingdom of the physician."

